# Insights into chloroplast genome structure and phylogenetic relationships within the *Sesamum* species complex (Pedaliaceae)

**DOI:** 10.3389/fgene.2023.1207306

**Published:** 2023-05-30

**Authors:** Yedomon Ange Bovys Zoclanclounon, Senthil Kumar Thamilarasan, Youngjun Mo, Byoung-Ohg Ahn, Jeong-Gu Kim, Keunpyo Lee

**Affiliations:** ^1^Genomics Division, National Institute of Agricultural Sciences, Rural Development Administration, Jeonju, Republic of Korea; ^2^ Department of Crop Science and Biotechnology, Jeonbuk National University, Jeonju, Republic of Korea

**Keywords:** phylogeny, chloroplast genome, *Sesamum*, *Ceratotheca*, wild relatives, species complex

## Abstract

**Background:** In the *Sesamum* species complex, the lack of wild species genomic resources hinders the evolutionary comprehension of phylogenetic relationships.

**Results:** In the present study, we generated complete chloroplast genomes of six wild relatives (*Sesamum alatum*, *Sesamum angolense*, *Sesamum pedaloides*, *Ceratotheca sesamoides* (syn. *Sesamum sesamoides*), *Ceratotheca triloba* (syn. *Sesamum trilobum*), and *Sesamum radiatum*) and a Korean cultivar, *Sesamum indicum* cv. Goenbaek. A typical quadripartite chloroplast structure, including two inverted repeats (IR), a large single copy (LSC), and a small single copy (SSC), was observed. A total of 114 unique genes encompassing 80 coding genes, four ribosomal RNAs, and 30 transfer RNAs were counted. The chloroplast genomes (152, 863–153, 338 bp) exhibited the IR contraction/expansion phenomenon and were quite conserved in both coding and non-coding regions. However, high values of the nucleotide diversity index were found in several genes, including *ndhA*, *ndhE*, *ndhF*, *ycf1*, and *psaC–ndhD*. Concordant tree topologies suggest *ndhF* as a useful marker for taxon discrimination. The phylogenetic inference and time divergence dating indicate that *S. radiatum* (2n = 64) occurred concomitantly with the sister species *C. sesamoides* (2n = 32) approximately 0.05 million years ago (Mya). In addition, *S. alatum* was clearly discriminated by forming a single clade, showing its long genetic distance and potential early speciation event in regards to the others.

**Conclusion:** Altogether, we propose to rename *C. sesamoides* and *C. triloba* as *S. sesamoides* and *S. trilobum*, respectively, as suggested previously based on the morphological description. This study provides the first insight into the phylogenetic relationships among the cultivated and wild African native relatives. The chloroplast genome data lay a foundation for speciation genomics in the *Sesamum* species complex.

## Introduction

The *Sesamum* L. genus belongs to the Pedaliaceae family with approximately 80 species grouped in 17 genera (Cronquist, 1981). Its leaves are alternate or opposite, and the inflorescence appears generally as a solitary type in leaf axils with the presence of extra-floral nectaries (Bedigian, 2015). The number of species in this genus is under constant revision, since the classification criteria were quite diverse according to the authors. The index Kewensis listed 34 species (Nayar and Mehra, 1970). Later on, Kobayashi (1991) reported 38 species. Through the construction of the *Sesamum* spp. descriptor, the number was revised to 20 species (IPGRI and NBPGR, 2004; Bedigian, 2015). Based on the Plants of the World Online database, a total of 31 species have been accepted, including 22 wild species native from Africa (POWO, 2022).

The wild relatives are mainly distributed across tropical Africa (from Senegal to Somalia), central and southern Africa, and in drought-prone Indian subcontinent areas (Bedigian, 2015). Both dietary habits and traditional medicine practices are marked by the usage of cultivated and wild relatives (Ntwenya et al., 2017; Aworh, 2018; Bedigian, 2018). Among the therapeutic virtues of sesame, lowering cholesterol is one of the important functions that were reported for preventing high blood pressure disease (Hsu and Parthasarathy, 2017). This function was imputed to the presence of singular lignans known as sesamolin and sesamin (Visavadiya and Narasimhacharya, 2008).

The progenitor and the domestication history underpinning the cultivated sesame have been a subject of debate. Despite the high number of wild relatives in Africa, the investigations based on the interspecific hybridization ability (Bedigian, 2014), the presence or absence of sesamolin (Bedigian et al., 1985; Bedigian, 2003), and external transcribed spacer-based phylogeny reconstruction (Gormley et al., 2015) suggested the Indian native wild *Sesamum malabaricum* as the probable progenitor. In addition, the scientific controversy relative to the center of origin of the cultivated sesame opposed Africa and the Indian subcontinent (Bedigian, 2003).

Moreover, several species belonging to the *Josephinia*, *Dicerocaryum*, and *Ceratotheca* genus were reported to be closely related to *Sesamum* species based on phenotypic data and limited number of plastid markers (*trnL*–*trnF* and *ndhF*) and external transcribed spacer sequences (Gormley et al., 2015). More specifically, these genera form a species complex with *Sesamum*, making it difficult to clearly delineate species boundaries (Manning and Magee, 2018).

The chloroplast organelle is referred to as a chemical factory of plant cells involved in the crucial metabolism of green plants known as photosynthesis (Kirchhoff, 2019). Due to its uniparental inheritance and non-recombination intrinsic characteristics, the chloroplast is widely used to infer the phylogenetic relationships at inter- and intra-taxon levels (Biju et al., 2019; Köhler et al., 2020; Zhou et al., 2020). To the best of our knowledge, only the *Sesamum indicum* taxon chloroplast genome has been assembled (Yi and Kim, 2012; Zhang et al., 2013). Due to the lack of wild relatives’ chloroplast genome, a comprehensive study on the phylogenetic relationship between *Sesamum* and *Ceratotheca* has not been elucidated yet. Interestingly, a total of three types of chromosome numbers (2n = 26, 2n = 32, and 2n = 64) have been reported in the *Sesamum* genus, and only 2n = 32 for *Ceratotheca* suggests a potential polyploidy or hybridization event occurrence (Raghavan and Krishnamurthy, 1947; Kobayashi, 1991; Patil and Hiremath, 2002; Patil and Hiremath, 2004). Therefore, a complete chloroplast genome offers a relevant opportunity to investigate the evolutionary relationship between the two sister species.

This study was designed to address two main questions: 1) what is the chloroplast genome organization variation between *Sesamum* and *Ceratotheca* representatives? 2) How phylogenetically related are the sister species? We took advantage of the whole-genome sequencing data 1) to assemble and annotate the first complete chloroplast genome of *Sesamum alatum*, *Sesamum pedaloides*, *Sesamum angolense*, *Sesamum radiatum*, *Ceratotheca sesamoides*, and *Ceratotheca triloba*; 2) to investigate their sequence polymorphism and divergence; and 3) to infer the phylogenetic relationships and time divergence among the species.

## Materials and methods

### Taxon sampling and DNA extraction

**FIGURE 1:**
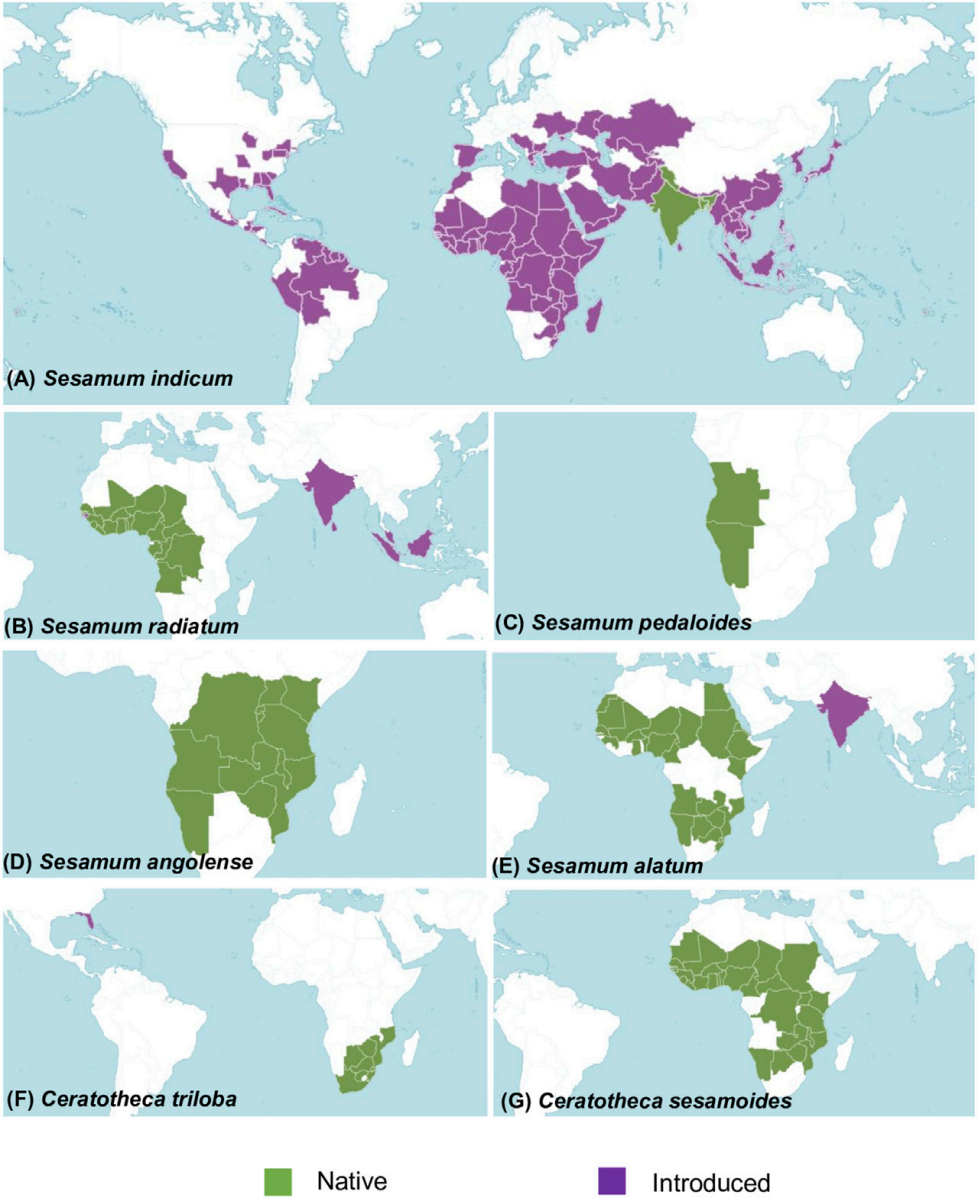
Map showing the geographical distribution of the studied species in their native (in green) and host habitat (in purple). **(A)** *Sesamum indicum* distribution. The native region covers Assam, Bangladesh, India, and western Himalaya. It has a worldwide distribution. **(B)** *Sesamum radiatum* is native to Angola, Benin, Burkina, Cameroon, Cape Verde, the Central African Republic, Chad, Congo, Gabon, the Gambia, Ghana, Guinea, the Gulf of Guinea Is., Ivory Coast, Liberia, Mali, Niger, Nigeria, Senegal, Sierra Leone, Togo, and the Democratic Republic of Congo. It has been introduced in Borneo, Guinea-Bissau, India, Malaya, Sri Lanka, and Sumatera. **(C)** *Sesamum pedaloides* is originated from Angola and Namibia. **(D)** *Sesamum angolense* came from the southern part of Africa covering Angola, Burundi, Kenya, Malawi, Mozambique, Namibia, Rwanda, Tanzania, Uganda, Zambia, the Democratic Republic of Congo, and Zimbabwe. **(E)** *Sesamum alatum* was introduced in India. Its native distribution covers Angola, Benin, Botswana, Burkina Faso, Cameroon, Caprivi Strip, Chad, Egypt, Eritrea, Ethiopia, Ghana, Guinea, Kenya, KwaZulu-Natal, Mali, Mauritania, Mozambique, Namibia, Niger, Nigeria, Senegal, Sudan, Swaziland, Western Sahara, Zambia, and Zimbabwe. **(F)** *Ceratotheca triloba* originated from Botswana, Cape Provinces, the Free State, KwaZulu-Natal, Mozambique, Swaziland, and Zimbabwe. It has been introduced in Florida (US). **(G)** *Ceratotheca sesamoides* is native to Benin, Botswana, Burkina, Cameroon, the Caprivi Strip, the Central African Republic, Chad, Gambia, Ghana, Guinea, Guinea-Bissau, Ivory Coast, Kenya, Liberia, Malawi, Mali, Mauritania, Mozambique, Namibia, Niger, Nigeria, Senegal, Sierra Leone, Sudan, Tanzania, Togo, Uganda, Zambia, the Democratic Republic of Congo, and Zimbabwe. Distribution data of each species were collected from POWO (2022).

A total of six wild sesame species, namely, *S. alatum* (2n = 26), *S. angolense* (2n = 32), *S. radiatum* (2n = 64), *S. pedaloides* (2n = indeterminate), *C. sesamoides*, and *C. triloba* were provided by the Korean genebank (Supplementary Table S1). The natural distribution of wild relatives is summarized in Figure 1. In addition, the Korean elite cultivar Goenbaek (2n = 26) was also employed for *de novo* chloroplast genome assembly. The seeds were grown under field conditions during the 2020 summer season (May–September) at the National Institute of Agricultural Sciences in Jeonju (35°49′50.37″N latitude, 127°3′52.79″E longitude).

Young leaves of each species were sampled, and their DNA was extracted following a modified CTAB protocol (Allen et al., 2006). Afterward, DNA purity was checked in 1% agarose gel (1× TAE) using the NanoDrop^®^ ND-1000 UV-vis spectrophotometer (Thermo Fisher Scientific, United States). The extracted DNA samples were stored at −20°C prior to further usage.

### Library preparation and sequencing

The TruSeq DNA Nano Library Preparation Kit (Illumina, San Diego, United States) was used to construct the library by fragmenting 1 µg high-quality genomic DNA of each sample followed by 5′ and 3′ adapter ligation. The NovaSeq 6000 machine (Illumina, San Diego, United States) served as a platform for short-read sequencing.

### Assembly and annotation

For *de novo* chloroplast assemblies, we used GetOrganelle with default parameters (Jin et al., 2020). The Rubisco subunit gene (GenBank accession: HQ384882.1) of the reference chloroplast genome (GenBank accession: NC_016433.2) from *S. indicum cv.* Ansanggae was provided as a seed.

All chloroplast assemblies were annotated with GeSeq (Tillich et al., 2017). The setting parameters were defined as follows: HMMER profile search for coding genes and ribosomal RNA annotation, ARAGORN v1.2.38 (Laslett and Canback, 2004) and tRNAscan-SE v2.0.5 (Chan and Lowe, 2019) for transfer RNA gene detection, and the *S. indicum* L. *cv.* Ansanggae chloroplast as a reference for the homology-based annotation purpose. Chloë v1.1, a stand-alone chloroplast annotator (https://chloe.plantenergy.edu.au/annotate.html), served as an additional third party annotator for comparison. Using the reference chloroplast annotation, pseudo-genes and trans-spliced genes were manually inspected. The chloroplast genome map was rendered using OrganellarGenomeDRAW (OGDRAW) version 1.3.1 (Greiner et al., 2019).

### Wet-lab validation of plastome junction sites

To check the quality of each assembly, an alignment to the reference chloroplast genome (GenBank accession: NC_016433.2) was conducted using the BLASTN tool (Altschul et al., 1990). Subsequently, the position of each chloroplast genome junction was detected. Thus, primers flanking the junction sites were designed using primer3 v2.3.6 (Untergasser et al., 2012), and a PCR-based validation was carried out under the conditions described as follows: the total volume of 20 μL encompassed 15 ng of DNA, 10 pmol of each primer, and the dried SafeDry Taq LTP-480 Premix (CellSafe Co., Ltd., Gyeonggi-do, Korea). PCR experiments were conducted in eight strip tubes in a C1000 Thermal Cycler (Bio-Rad, Hercules, CA, United States). PCR cycles were 95°C (3 min) and 35 cycles at 95°C (30 s), 55°C (30 s), and 72°C (30 s), followed by the extension step for 5 min at 72°C. The amplified products were separated in 1% agarose gel (1× TAE) and visualized using a UVP GelSolo M-26XV imager (Analytik Jena, CA, United States).

### Comparative chloroplast genome analysis

The annotated chloroplast genomes were compared using the mVISTA web server (http://genome.lbl.gov/vista/mvista/submit.shtml) (Frazer et al., 2004), with the cultivar Ansanggae chloroplast genome as a reference. Shuffle-LAGAN was selected as the alignment mode. In order to identify putative gene rearrangements or synteny patterns within the chloroplast genomes, a whole-genome alignment was executed in AliTV (Ankenbrand et al., 2017) and Mauve v.2.4.0 with the progressiveMauve algorithm option (Darling et al., 2004), respectively.

The IR/LSC and IR/SSC boundaries of the chloroplast genomes were visualized using the IRscope R Shiny web app (https://irscope.shinyapps.io/irapp/) (Amiryousefi et al., 2018). The *Arabidopsis thaliana* chloroplast genome (GenBank accession: NC_000932.1) was included as an outgroup. The nucleotide diversity (π) among the assembled chloroplast genomes was calculated using DnaSP v.6.12.3 (Rozas et al., 2017).

### Repeat analysis

Palindrome, complement, forward, and reverse sequence identification was carried out using the REPuter web server (https://bibiserv.cebitec.uni-bielefeld.de/reputer) (Kurtz et al., 2001). The minimal repeat size and hamming distance were set to 30 bp and 3, respectively. Simple sequence repeats (SSRs) were identified using the MISA-web program (https://webblast.ipk-gatersleben.de/misa/) with default parameters (Beier et al., 2017).

### Phylogenetic inference

To infer the phylogeny among *Sesamum*, *Ceratotheca*, and close members from the Lamiales order, chloroplast genome datasets (Supplementary Table S2) were retrieved from the NCBI. *Vitis vinifera* was used as an outgroup. A total of 75 common protein-coding genes (Supplementary Table S3) served as the phylogeny inference. A multiple sequence alignment was performed using MAFFT v7.471-0 (Katoh and Standley, 2013). The multiple sequence alignment files were trimmed using trimAl (Capella-Gutierrez et al., 2009) to remove poorly aligned regions. Afterward, the maximum-likelihood tree was constructed using IQ-TREE v2.0.3 (Nguyen et al., 2015), following the automatically selected best-fit model. The ultrafast bootstrap method (Minh et al., 2013) with 1,000 iterations was applied. Bayesian inference was also employed to infer the phylogenetic relationship using MrBayes v3.2.6 (Huelsenbeck and Ronquist, 2001).

### Divergence time estimation

To estimate the divergence time among the studied species, the RelTime method and the general time reversible model were performed in MEGA X, following the procedure defined by Mello (2018). Based on the TimeTree database (Kumar et al., 2017), two calibration constraints were set: 1) Pedaliaceae versus Acanthaceae: 34–70 Mya and 2) Linderniaceae versus Pedaliaceae: 41–66 Mya.

### Selection pressure analysis

The non-synonymous-to-synonymous substitution ratio (Ka/Ks), for each orthologous pair (with *S. indicum* as reference), was computed using the codeml package from the PAML tool (Yang, 1997). Prior to the calculation, a codon-based nucleic acid alignment was obtained from the initial protein-coding multiple sequence alignment file using PAL2NAL (Suyama et al., 2006). For a reliable interpretation, the ratios with Ks <0.01 or Ks >2 were filtered out.

### Codon usage bias and RNA editing site analyses

Relative synonymous codon usage (RSCU) bias analysis was conducted using CodonW v1.4.4 (http://codonw.sourceforge.net/, accessed 12 February 2021). The PREP-Cp package from the PREP suite (Mower, 2009) was used to predict RNA editing sites for each species, with a cut-off value of 0.8.

## Results

### General features of the assembled chloroplast genomes

**FIGURE 2:**
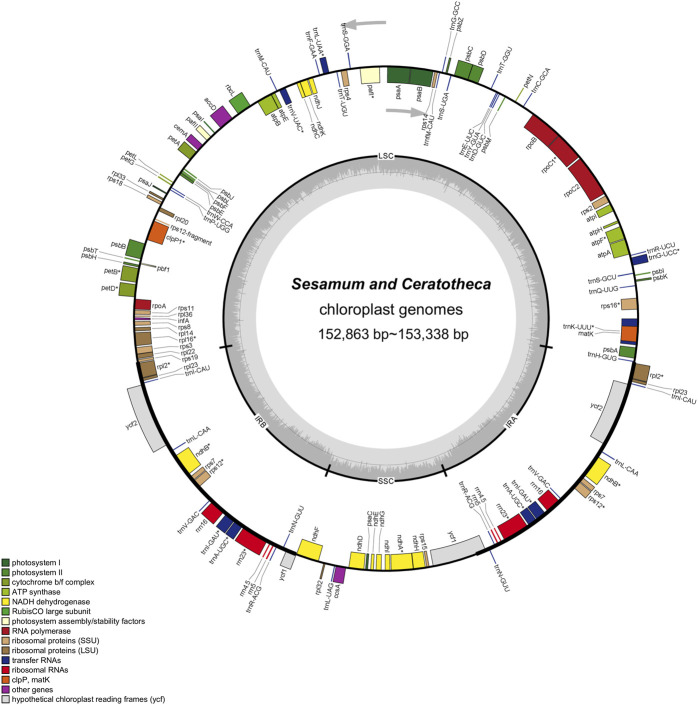
Chloroplast genome map of *Sesamum* and *Ceratotheca* species. Intron-containing genes are marked with the asterisk symbol (*). The dashed gray area in the inner circular map indicates the GC content. Different functional gene categories are colored as indicated in the lower part of the genome map.

The assembled chloroplast genomes resulted in a single circular quadripartite genome with two typical IRs separated by LSC and SSC (Figure 2). The plastome sizes were 153,089 bp, 153,096 bp, 153,217 bp, 153,285 bp, 153,338 bp, 153,287 bp, and 152,863 bp for *S. angolense*, *S. alatum*, *S. pedaloides*, *S. radiatum*, *S. indicum cv.* Goenbaek, *C. sesamoides*, and *C. triloba*, respectively (Table 1). The seven genomes contained 114 unique genes, including 80 coding proteins, 30 transfer RNAs, and four ribosomal RNA genes, as previously found by Yi and Kim (2012) and Zhang et al. (2013) using *S. indicum cv.* Ansanggae and *S. indicum cv.* Yuzhi 11 as plant models, respectively. Among the assembled chloroplast genomes, 10 coding sequence genes (*atpF*, *rpoC1*, *rps12*, *petB*, *petD*, *rps16*, *rpl2*, *rpl16*, *ndhA*, and *ndhB*) harbor a single intron, while two coding genes (*clpP* and *ycf3*) contain two introns. The GC contents were similar in IR (43.39% ± 0.01%), LSC (36.40% ± 0.03%), SSC (32.62% ± 0.08%), and whole-chloroplast genome scales (38.26% ± 0.03%) (Table 1).

**TABLE 1:** Complete chloroplast genome statistics of the wild and cultivated sesame species.

	*Sesamum indicum cv.* Goenbaek	*Sesamum alatum*	*Sesamum angolense*	*Sesamum pedaloides*	*Sesamum radiatum*	*Ceratotheca sesamoides*	*Ceratotheca triloba*
Plastome length (bp)	153,338	153,096	153,089	153,217	153,285	153,287	152,863
GC content (%)	38.2	38.27	38.25	38.26	38.27	38.27	38.29
IR length (bp)	25,142	25,150	25,157	25,190	25,131	25,131	25,096
IR GC content (%)	43.39	43.42	43.37	43.39	43.39	43.39	43.41
LSC length (bp)	85,180	85,004	85,106	85,118	85,183	85,185	84,872
LSC GC content (%)	36.34	36.38	36.38	36.41	36.43	36.43	36.44
SSC length (bp)	17,874	17,792	17,669	17,719	17,840	17,840	17,799
SSC GC content (%)	32.47	32.75	32.67	32.59	32.63	32.64	32.62
CDS count	80	80	80	80	80	80	80
tRNA count	30	30	30	30	30	30	30
rRNA count	4	4	4	4	4	4	4
Gene count	114	114	114	114	114	114	114

### Phylogenetic relationships between *Sesamum* and *Ceratotheca* species

**FIGURE 3:**
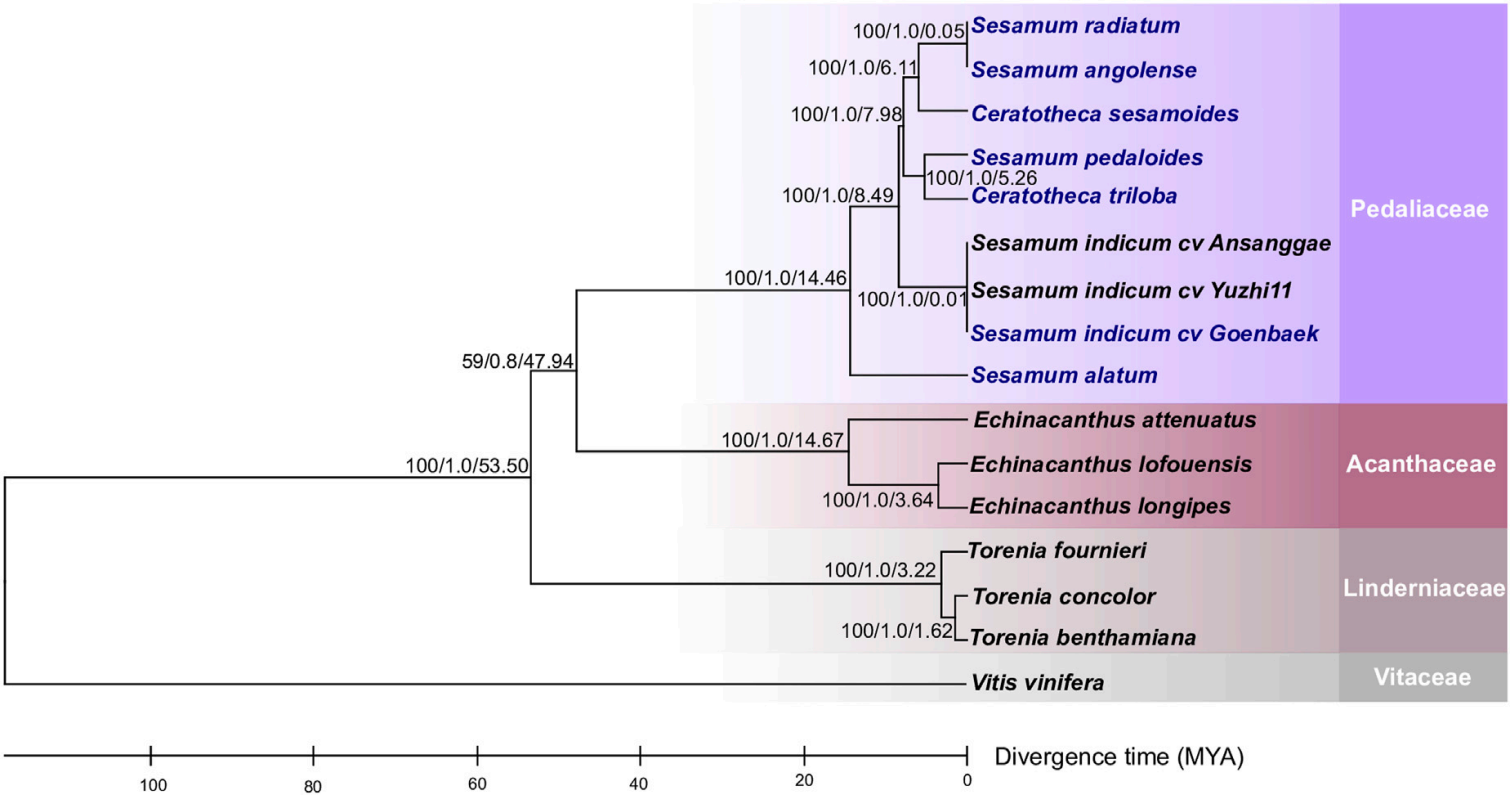
Phylogeny placement of newly constructed chloroplast genomes within the Lamiales members. The phylogenetic tree was inferred from the maximum-likelihood and Bayesian analyses of 75 conserved coding genes. The closest Lamiales species cover Acanthaceae and Linderniaceae species. *Vitis vinifera* was set as an outgroup. The numbers above the nodes are support values with maximum-likelihood bootstrap values on the left, Bayesian bootstrap values in the middle, and divergence times (in millions of years) estimated using the RelTime approach implemented using MEGA X software. The newly assembled chloroplast genomes are indicated in blue.

To figure out the evolutionary relationship among *Sesamum*, *Ceratotheca*, and the closest related species belonging to Acanthaceae and Linderniaceae, a tree (Figure 3) was constructed using 75 shared protein sequences. *Vitis vinifera* was employed as an outgroup. As expected, *Sesamum* and *Ceratotheca* representatives clustered in a single clade, resolving the sister species relationship. A close view of the Pedaliaceae clade highlighted four major sub-clades. The first sub-clade represents a mixture of two ploidy levels with *S. radiatum* (2n = 64), *S. angolense* (2n = 32), and *C. sesamoides* (2n = 32). Interestingly, the second sub-clade encompassed *S. pedaloides* (2n = unknown) and *C. triloba* (2n = 32), suggesting that the ploidy of *S. pedaloides* might also be 2n = 32. The cultivated species *S. indicum* (2n = 26) is grouped into one sub-clade, while the wild relative *S. alatum* (2n = 26) constitutes a single sub-clade as being genetically distant from the cultivars and other wild relatives.

### Divergence time estimation

In order to understand the speciation occurrence time among *Sesamum* and *Ceratotheca* species, a time divergence analysis was performed (Figure 3). First, *S. alatum* was estimated to have occurred 14.46 million years ago (Mya). Second, *S. pedaloides* and *C. triloba* split concomitantly approximately 5.26 Mya. Third, *C. sesamoides* was inferred to have occurred 6.11 Mya, a little bit earlier than *S. pedaloides* and *C. triloba*. As expected, the cultivated type (*S. indicum*) occurred lastly at 0.01 Mya. Interestingly, the time divergence inference revealed that *S. radiatum* (2n = 64) and *C. sesamoides* (2n = 32) have recently concomitantly occurred at approximately 0.05 Mya.

### Comparative plastome analysis

**FIGURE 4:**
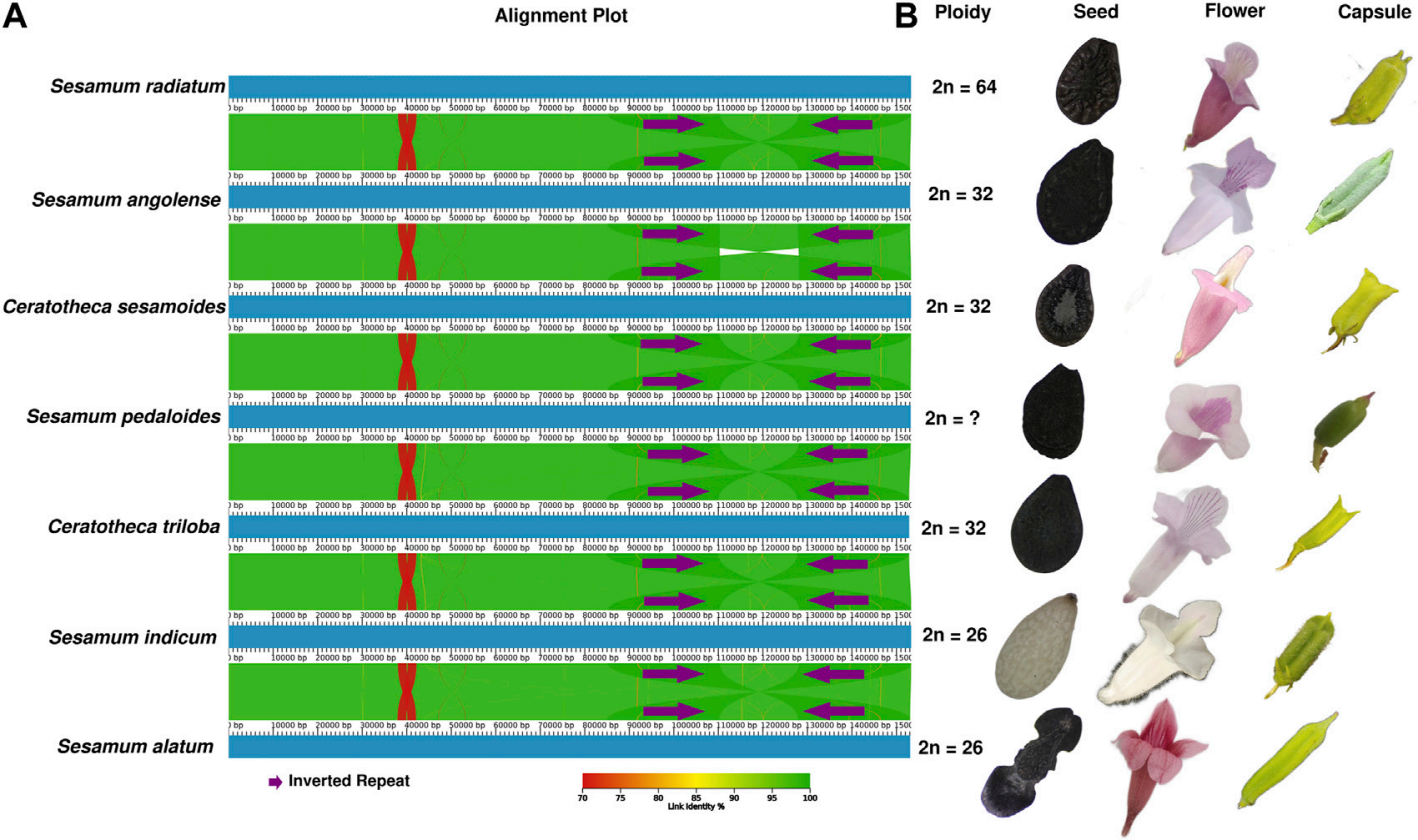
Morphological and plastome-based comparative analyses between *Sesamum* and *Ceratotheca* species. The alignment view of chloroplast genomes using the all-vs-all alignment approach implemented in AliTV software **(A)**. The purple arrows represent the IR region. Each genome is colored following the link identity percentage as shown in the right corner. The ploidy, seed capsule, and flower photos were added to the figure **(B)**.

The chloroplast genome structure is highly conserved (Figure 4A), although the morphological features of the species are distinct (Figure 4B). When comparing the plastid genome structure using mVISTA (Shuffle-LAGAN) (Supplementary Figure S1 **)**, Mauve (progressiveMauve alignment) (Supplementary Figure S2), and AliTV (LASTZ all-vs-all alignment) (Figure 4A), the conservative genome structure in coding and non-coding regions was also confirmed. However, IR contraction and expansion has been revealed with a length ranging from 25,096 bp to 25,190 bp, while LSC varied from 84,872 bp to 85,185 bp. SSC was sized from 17,719 bp to 17,874 bp.

**FIGURE 5:**
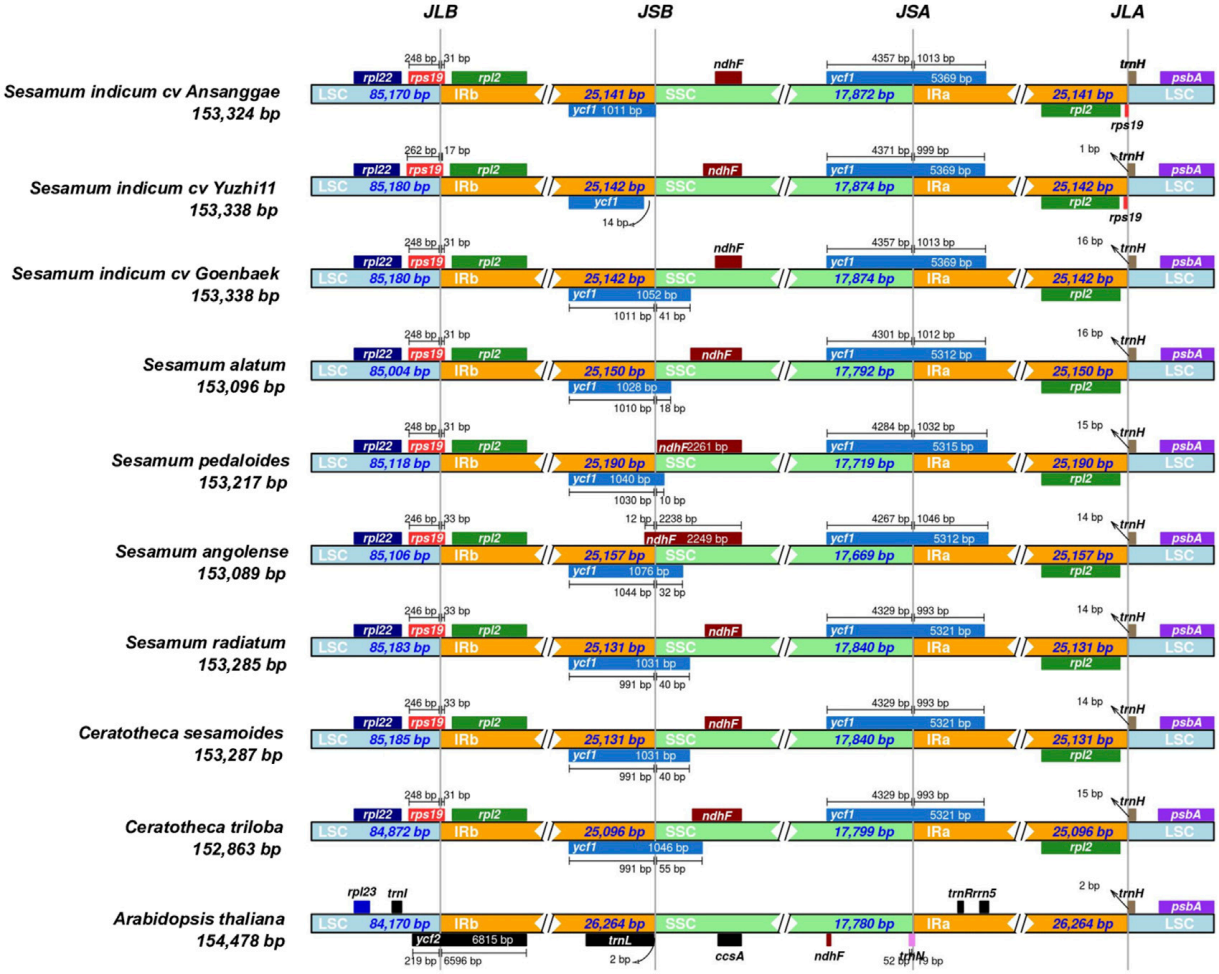
Chloroplast junction site view showing gene distribution alongside the boundaries and IR contraction and expansion within the *Sesamum* and *Ceratotheca* species. *Arabidopsis thaliana* was set as the outgroup.

By comparing chloroplast genome boundaries of *Sesamum* and *Ceratotheca* species, we noted that the IRb/LSC junction is sited between *rpl2* and *rsp19* genes (Figure 5). The pseudogene *ycf1* is located exclusively in IRb for *S. indicum cv.* Ansanggae, *S. indicum cv.* Yuzhi 11, and at the border of the IRb/SSC of other species. The *ndhF* gene is mainly found in the SSC region for all species, except for *S. angolense*. The *ycf1* gene of all species was located at SSC/IRa junctions with a gene size ranging from 5,312 to 5,369 bp. The *trnH* gene was localized in the LSC region, 1–16 bp away from the IRa–LSC border. Overall, the chloroplast genome structure at different junctions was highly conserved among *Sesamum* and *Ceratotheca* species.

### Variation hotspots within chloroplast genomes of *Sesamum* and *Ceratotheca* species

**FIGURE 6:**
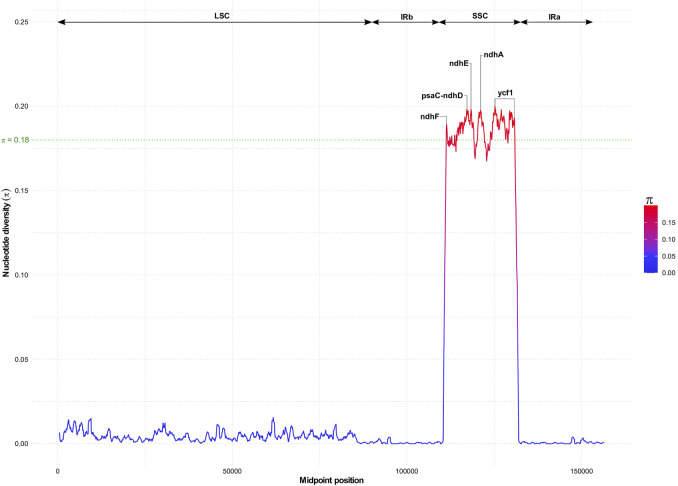
Nucleotide diversity variation with chloroplast genomes of *Sesamum* and *Ceratotheca* species. Highest values above 0.18 (in green) indicate candidate genes for the population genetics purpose.

**FIGURE 7:**
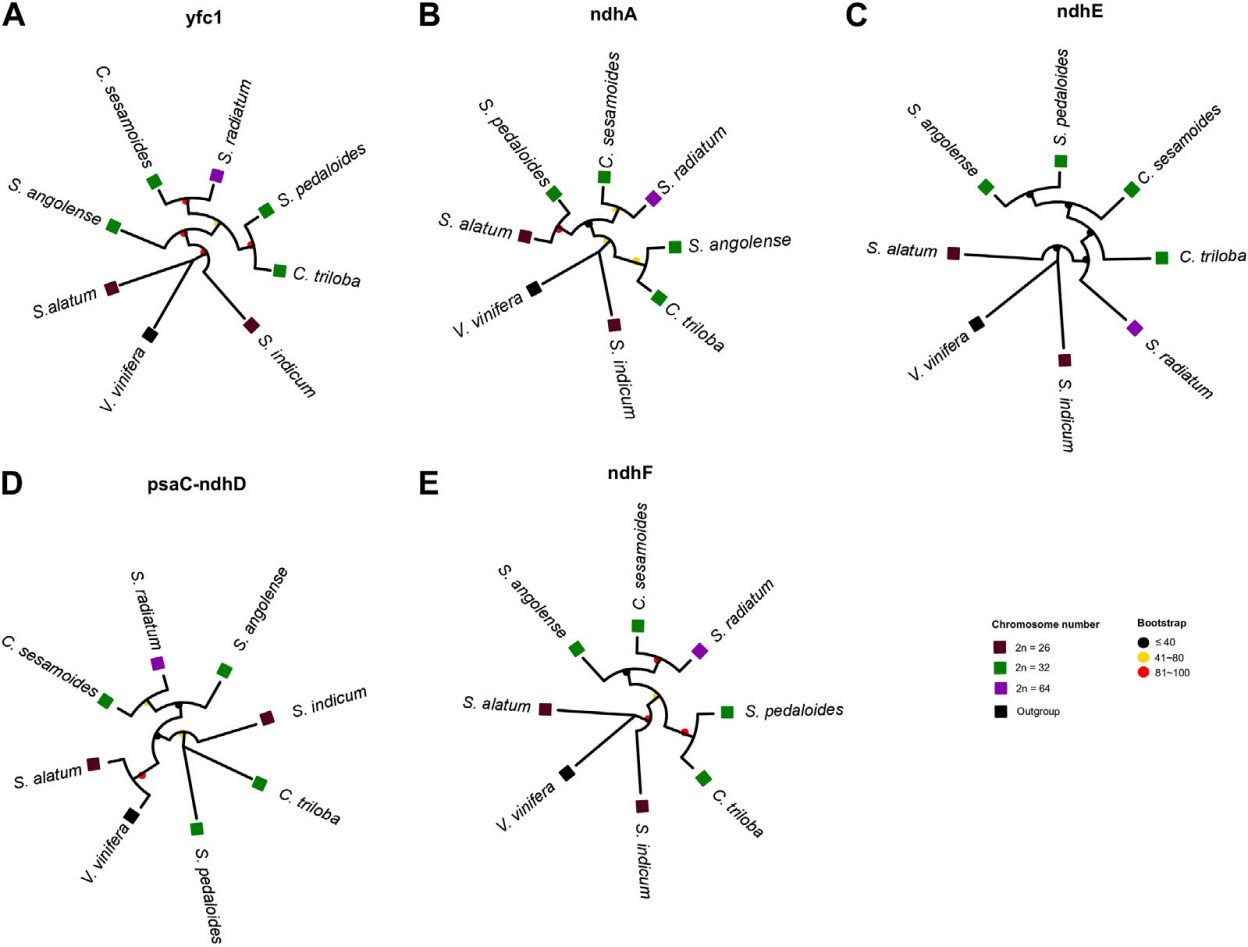
Evaluation of the discriminatory power of candidate regions, namely *ycf1* **(A)**, *ndhA* **(B)**, *ndhE* **(C)**, *psaC-ndhD* **(D)**, and *ndhF* **(E)**. The tree topology of *ndhF* **(E)** was consistent with the maximum-likelihood phylogenetic tree based on 75 coding genes. Discrepancies were noted for other gene tree inferences. *Vitis vinifera* was set as an outgroup. Squares and circles were colored following the ploidy and bootstrap values, respectively.

Despite the high collinearity of the chloroplast genome within *Sesamum* and *Ceratotheca* species, substantial variations were noted mainly in SSC regions (Figure 6). The nucleotide diversity calculation revealed a peak value located in *ycf1*, followed by *ndhA*, *ndhE*, *psaC*–*ndhD*, and *ndhF* regions. To estimate their discriminatory power, we inferred the phylogenetic tree using each gene (Figures 7A–E). As a result, only *ndhF* clearly distinguished the taxa (Figure 7E), as depicted previously with a set of 75 (Figure 3) protein-coding genes. Therefore, *ndhF* could be used as a marker to delineate *Sesamum* and *Ceratotheca* species, since several species are not yet well characterized at both morphological and cytogenetic levels.

### Codon usage bias analysis

Codon usage bias was examined by computing the RSCU (Sharp and Cowe, 1991). The RSCU represents the observed frequency of a codon divided by the expected frequency. A lack of bias referred to codons with RSCU values close to 1. Globally, a slight variation in the RSCU was found within *Sesamum* and *Ceratotheca* species (Supplementary Figure S3) values among *Sesamum* and *Ceratotheca* species. A total of 27 core codons exhibited RSCU >1, of which 24 were adenine/thymine-ending codons, one guanine-ending codon, and two cytosine-ending codons. In contrast, guanine- or cytosine-ending codons mostly exhibited RSCU <1. The most biased codon was found for the stop codon TAA (RSCU = 1.55 ± 0.01), while a less biased codon was detected for the stop codon TAG (RSCU = 0.69 ± 0.01). A similar trend of A–T bias in codon usage has been observed in other plant species (Wang et al., 2017; Biju et al., 2019).

Interestingly, by examining the species cluster from the heat map, the phylogenetic tree topology is concordant with the coding sequence-based tree inference, indicating a robust estimation of the phylogenetic relationship based on codon usage, as observed in a wide range of families (Gao et al., 2019; Chi et al., 2020; Wu et al., 2021).

### Long and simple sequence repeats

Long repeats constitute a driving force for chloroplast genome rearrangement and have been used for phylogenetic inferences between species (Park et al., 2017; Luo et al., 2021). They induce genetic diversity by promoting intermolecular recombination in the chloroplast genome (Park et al., 2018). Long repeats encompass forward, reverse, palindrome, and complement types. In the present study, the mean count of long repeats was 25.71 ± 3.24 bp. The number of long repeats ranged from 21 to 31, among which palindromic (12–18) and forward (9–13) types were the most abundant. Moreover, the size of the repeats was mainly within the range of 30–39 bp. Only *S. indicum* exhibited repeats in the range of 60–69 bp (Supplementary Figure S4).

Microsatellites are referred to short tandem repeat sequences of one to six nucleotide repeats (Fan and Chu, 2007). SSRs are widely present in the chloroplast genome and have been extensively used as molecular markers for population genetics, phylogenetic relationship inferences, and species identification (Powell et al., 1995; Huang et al., 2018; Lee et al., 2019; Li et al., 2020). We detected 21–32 chloroplast SSRs within the assembled chloroplast genomes (Supplementary Figure S5A; Supplementary Table S4), of which most are monomeric (>87%) (Supplementary Figure S5B). The majority (13–25) of SSRs are located in LSC sequences (Supplementary Figure S5C) and mainly in intergenic regions (>76%) for all species (Supplementary Figure S5D). The most dominant motif (A) count ranged from 19 to 27 and spanned 208–277 bp (Supplementary Figure S6). Trinucleotide repeats (AAT) were only detected for *S. alatum* occupying 30 bp of the chloroplast genome length (Supplementary Figure S6).

### Selection pressure analysis

The pairwise ratio of non-synonymous substitutions (Ka) to the rate of synonymous substitutions (Ks) analysis is presented in Supplementary Figure S7. Ka/Ks ratios with Ks <0.1 or K >0.2 were changed to zero for a reliable estimation of the selection pressure. The results revealed that the NAD(P)H-quinone oxidoreductase subunit I (*ndhI*) has undergone strong positive selection in *S. angolense* and *S. radiatum*. Similar trends were observed for the photosystem I gene *ycf4*, mainly in *S. radiatum*, *S. alatum*, and *C. sesamoides*. The *rpl20* gene also exhibited positive selection only in *S. alatum*. However, *matK*, *ndhF*, and *ycf1* Ka/Ks values were approximately equal to 1, implying neutral selection pressure.

### RNA editing analysis

RNA editing is an important biological phenomenon that plays a crucial role in the regulation of gene expression, the diversification of proteins, and adaptation to environmental changes (Mohammed et al., 2022). From the seven chloroplast genomes, we predicted 140 ± 5 RNA editing sites, which were found in 27 ± 1 genes (Supplementary Tables S5–S11). The *psaB* gene contained the highest number (16) of editing sites within the species. Meanwhile, the *rpoB* gene was predicted to have an average of 14 editing sites, while *ndhD* and *ndhF* genes exhibited 10 editing sites, respectively. The abundant editing site conversion within species was C-to-U transition, which is primarily found in a majority of plant chloroplast genomes (Hao et al., 2021). Globally, the change from proline to leucine was most frequent followed by thymine to inosine, proline to serine, and serine to leucine (Supplementary Tables S5–S11).

## Discussion

### Plastome evolution between the *Sesamum* and *Ceratotheca* genus

We reported for the first time, whole-chloroplast genomes of six African native wild sesame species: *S. alatum*, *S. angolense*, *S. pedaloides*, *S. radiatum*, *C. sesamoides*, and *C. triloba*. The information from the generated plastome sequences served as the basis for comparative analyses. A typical quadripartite chloroplast structure, including LSC, SSC, and two IRs, was observed. In-depth comparative analyses revealed contraction and expansion events within all species. IR expansion and contraction is a common phenomenon observed in land plants resulting in the variation of chloroplast length at both intra- and inter-species levels (Asaf et al., 2020; Guo et al., 2021). As expected, the chloroplast genome was highly conserved among *Sesamum* and *Ceratotheca*, despite the morphological differences. The conserved structure is consistent with the two previously published *S. indicum* chloroplast genomes (Yi and Kim, 2012; Zhang et al., 2013).

The variation of microsatellite copy numbers in the chloroplast genome is helpful for population genetics and polymorphism assessment. For instance, microsatellites with one nucleotide motif dataset provided in the current study constitute a useful resource for further population polymorphism assessment within *Sesamum*, *Ceratotheca*, and potentially close relative species in Pedaliaceae. Palindromic repeats are known to constitute mutational hotspots contributing to plastome expansion (Smith, 2020). By mining the chloroplast genomes, we detected that palindromic repeats are prominent in all chloroplast genomes. Therefore, they represent a suitable resource for marker development in regard to genetic diversity investigation in the *Sesamum* species continuum.

Chloroplast genes generally evolved under purifying selection, mainly to maintain the functional continuity of genes over a long period of time (Matsuoka et al., 2002; Jiang et al., 2018). However, previous comparative plastome studies identified some genes that underwent positive selection, including the photosynthetic genes *rbcL* (Ivanova et al., 2017) and *ycf2* (Jiang et al., 2018), among others.

In our study, mainly photosynthesis-related genes including *ndhA*, *ndhI*, and *ycf4* exhibited strong positive selection. Subsequently, owing to the specific distribution patterns of the studied samples in tropical Africa (See Figure 1) and the drought-prone habitat preference in nature, we postulate that the selection of this category of genes might be related to adaptation to environmental changes including the photosynthetic rate, drought, temperature, carbon dioxide level, or ecological niche (Piot et al., 2018). Moreover, the capability of photosynthetic-oriented gene selection may contribute to the drought tolerance strength of the cultivated sesame *S. indicum*, as revealed by previous extensive functional genomic studies (Dossa et al., 2019; Yu et al., 2019).

### A high-resolution view for the delineation of Pedaliaceae species in the sesame speciation continuum

Delineating species is a challenging aspect in taxonomy, since the methodology is quite heterogeneous depending not only on the taxa but also on the scientists. The Pedaliaceae s.l. family encompasses several tribes, including Sesamothamneae Ihlenf., Sesameae (Endl.) Meisn., and Pedaliae Dumort. Using plastid and nuclear markers, Gormley et al. (2015) provided evidence of the monophyletic pattern of these tribes. However, the authors highlighted that *Sesamum* is paraphyletic in regards with the *Ceratotheca*, *Josephinia*, and *Dicerocaryum* genus. This latter observation is in contrast with our results that showed that *Sesamum* and *Ceratotheca* formed a complex. The low number of markers and the used taxa in the previous study might explain the divergence of the tree topology. In fact, there were the absence of 2n = 64 chromosome set representatives. Therefore, our study provided the first insight regarding the chromosome number variation criterion.

The chloroplast genome is generally well conserved in land plants (Trösch et al., 2018). Despite the highly conserved chloroplast genome arrangement within both genera, remarkable sequence divergence was noted in several genes, including *ndhF* gene. The *ndhF*-based tree topology was consistent with the protein-coding gene tree, confirming that *ndhF* is a powerful candidate gene with promising potential for the DNA barcoding purpose.

Interestingly, a relatively long branch of *S. alatum* was observed implying its long evolutionary occurrence compared to that of the other species, which is consistent with the tree topology from Gormley et al. (2015), biogeographical and morphological data (Bedigian, 2018). It is noteworthy that *S. alatum* seeds exhibit a singular characteristic with winged-seeds, which is absent in the other species. The presence of wings is one of the key ecological adaptive trait of ancient wild crops that ensures seed dispersal by wind (Willson and Traveset, 2000). To the best of our knowledge, this trait seems to have been lost during evolution in the *Sesamum* genus, since no other member (described so far) of the genus harbors it. Moreover, the time divergence estimation revealed that *Sesamum alatum* diverged earlier (14.46 Mya) than other species, supporting the previous findings.

### Implications of the systematic placement of *Ceratotheca* in the *Sesamum* tribe (*Sesameae*)

In the *Sesamum* tribe, the classification of species has evolved following the descriptor. From the chloroplast genome analyses, we postulate that the nomenclature *C. sesamoides* and *C. triloba* might change into *Sesamum sesamoides* and *Sesamum trilobum* as suggested by Bruce (1953) and POWO (2022). Consequently, the section *Ceratotheca* (Endl.) J.C. Manning and Magee might be merged into the section *Sesamum*.

## Conclusion

The chloroplast genomes were highly conserved with respect to gene orientation, GC content, and gene content. However, the divergent sequences within species were detected in *ycf1*, *ndhA*, *ndhE*, and *ndhF* coding genes. The tree topology showed that *Sesamum* and *Ceratotheca* species were confidently resolved as sister species. Ultimately, the chloroplast sequence data from this study lay the foundation for the development of DNA barcoding markers and species-centered genomic research.

## Data Availability

The datasets presented in this study can be found in online repositories. The names of the repository/repositories and accession number(s) can be found in the article/Supplementary Material.
