# Supplementary material for: Insights into chloroplast genome structure and phylogenetic relationships within the Sesamum species complex (Pedaliaceae)
Source: Front Genet. 2023 May 30;14:1207306. doi: 10.3389/fgene.2023.1207306 (PMC10267711; doi:10.3389/fgene.2023.1207306)
Supplement: Supplementary file 1 [file DataSheet2.docx]

Supplementary Material

#
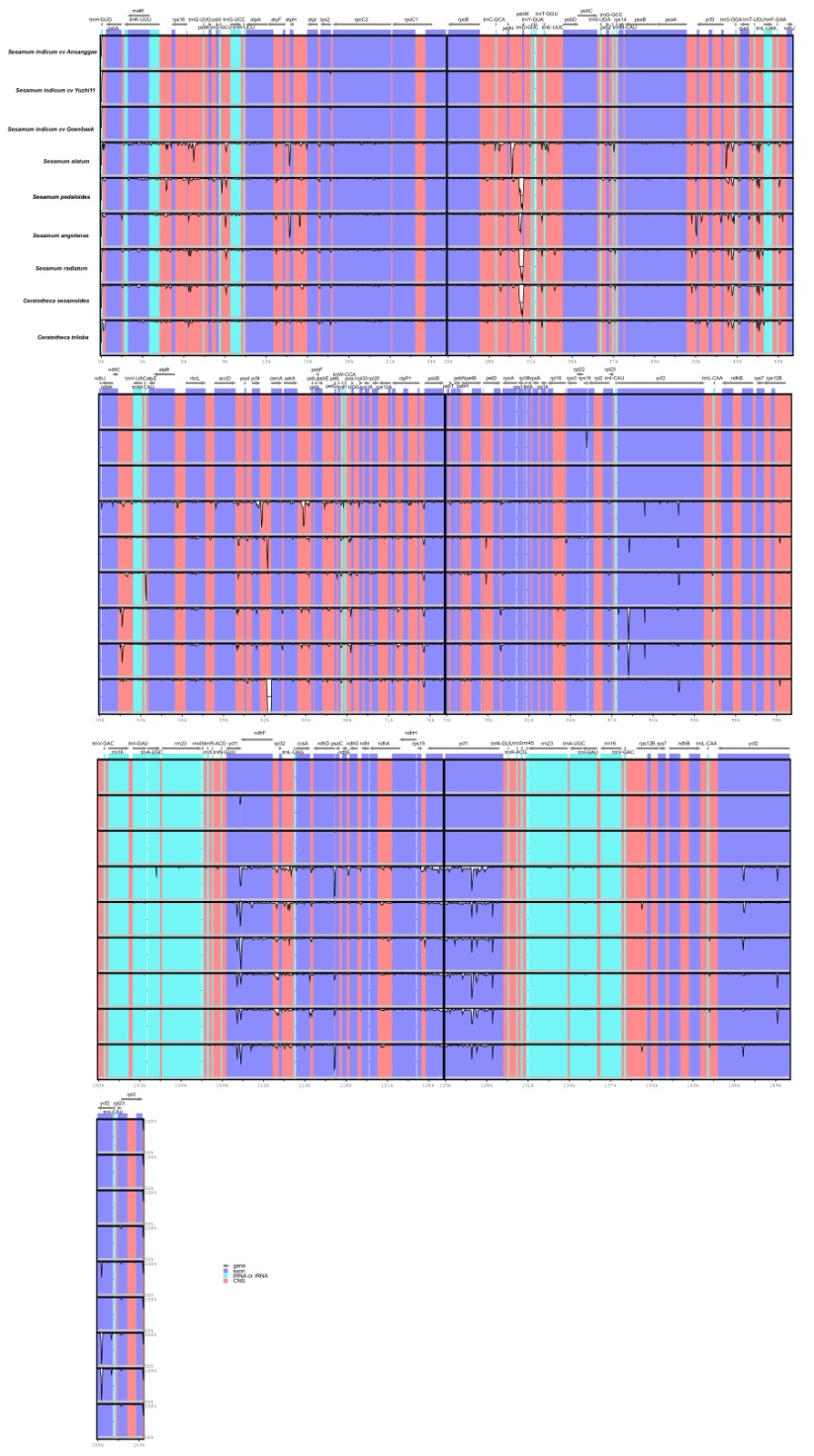
Supplementary Figures

**Supplementary Figure S1**. mVista view of the chloroplast alignment of seven *Sesamum* and two *Ceratotheca* species. The chloroplast genomes within the species are well conserved in both coding and non-coding regions.


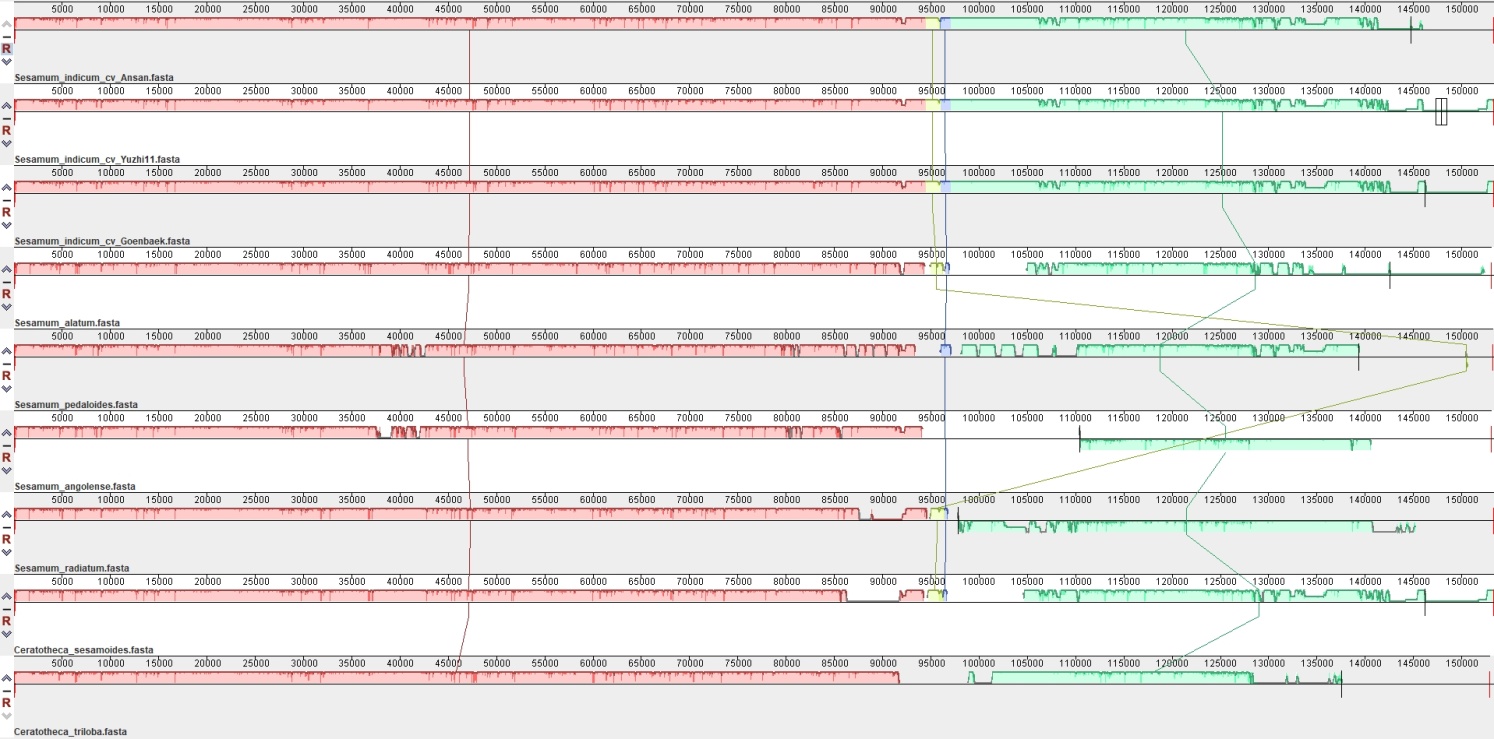


**Supplementary Figure S2.** Chloroplast genome alignment following progressive mauve alignment approach showing the conserved and divergent regions. The regions harboring the same color exhibit high similarities.

**Supplementary Figure S3**. Heat map of relative synonymous codon usage (RSCU) values among *Sesamum* and *Ceratotheca* species.
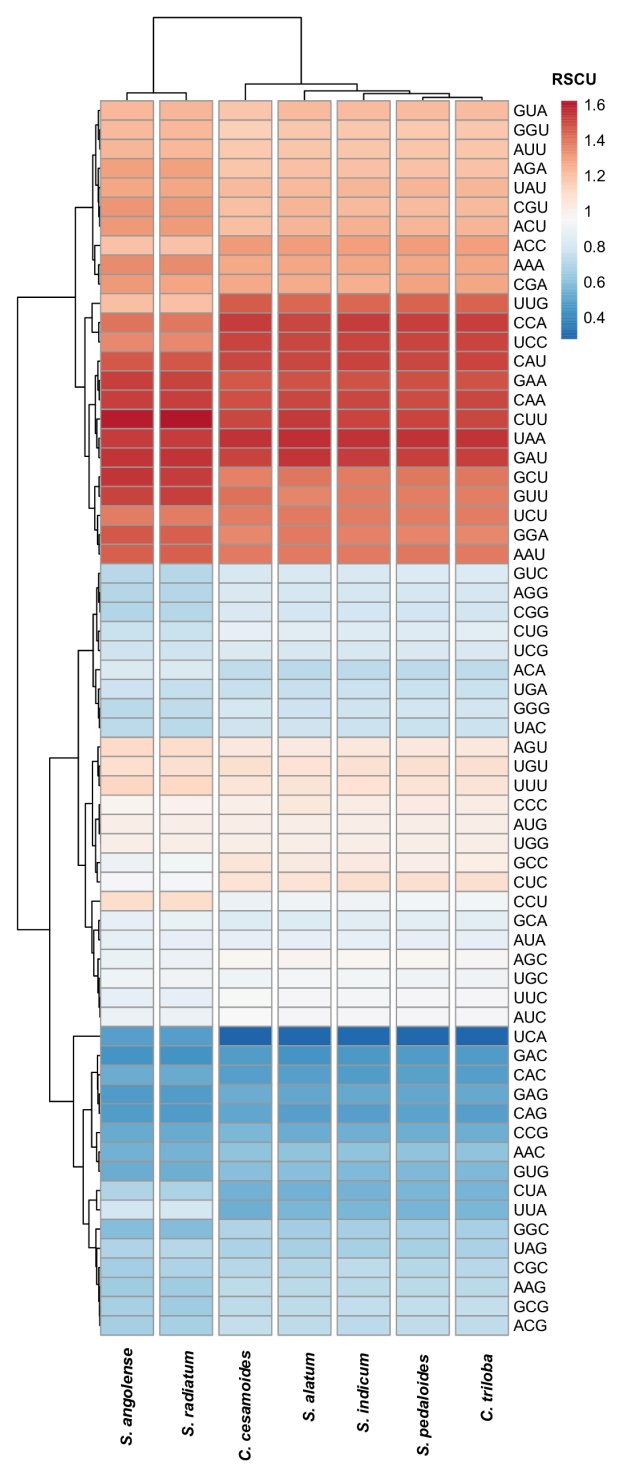


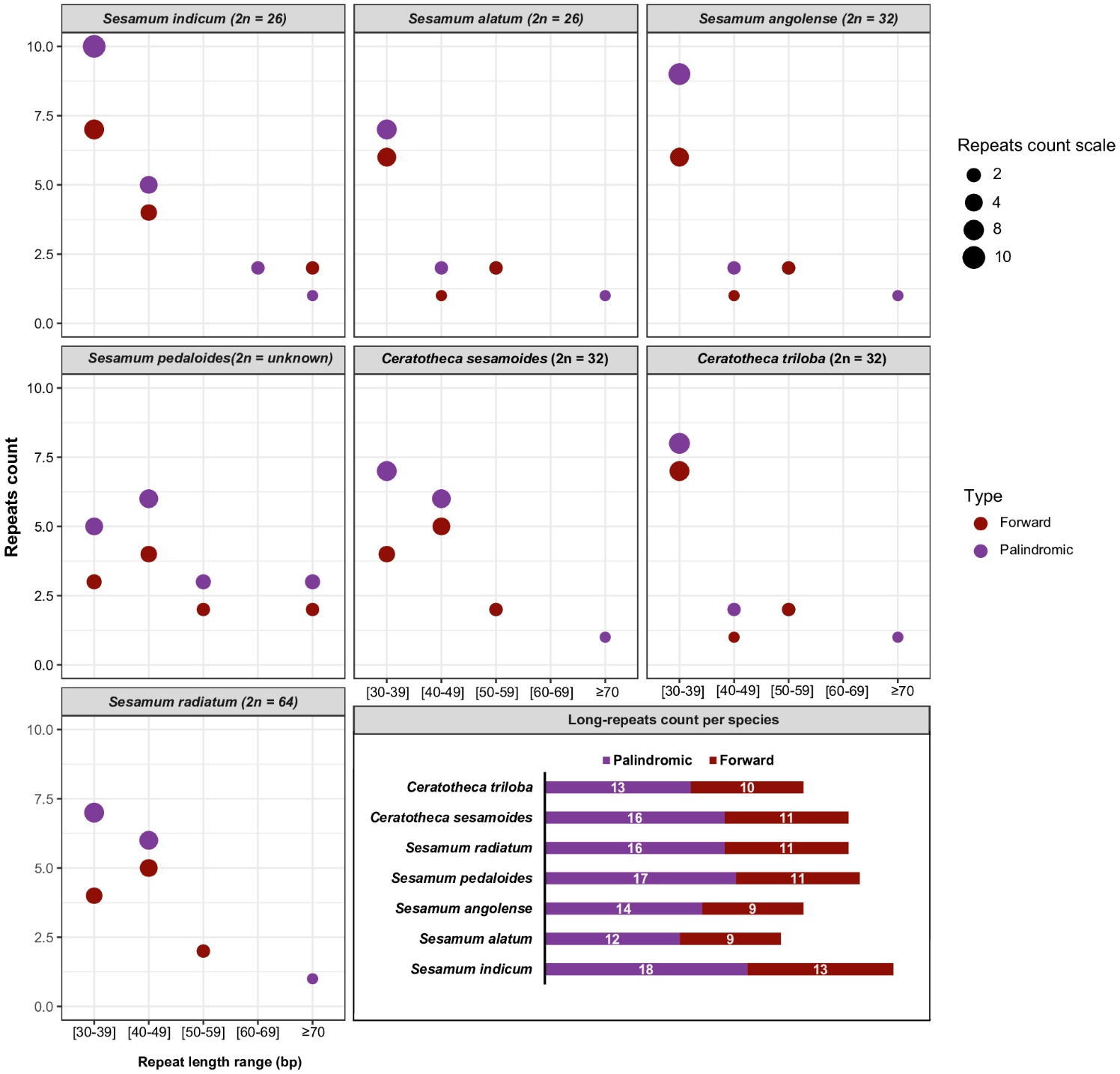


**Supplementary Figure S4**. Long-repeats count within Sesamum and Ceratotheca species chloroplast genomes.

#
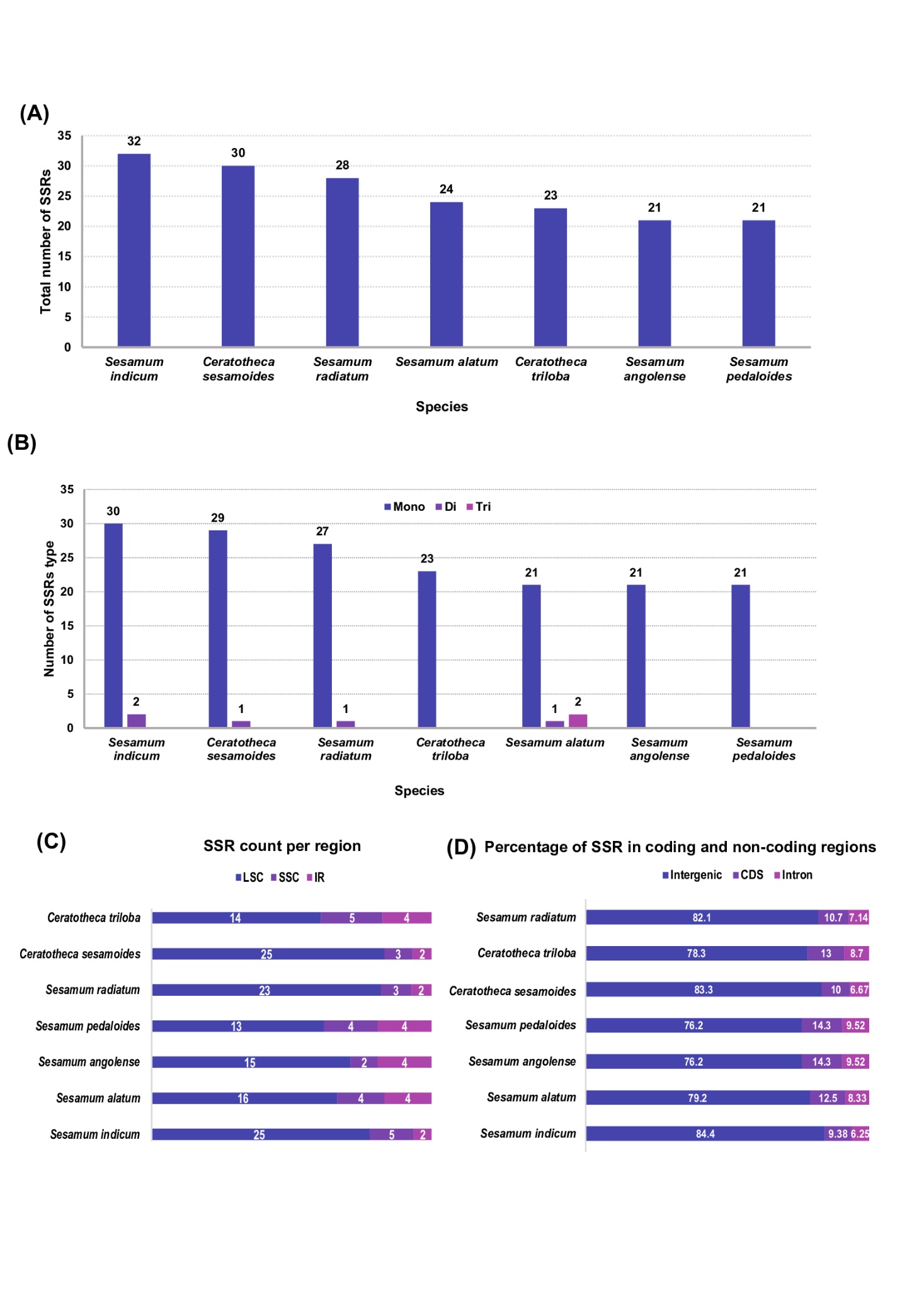


**Supplementary Figure S5**. Microsatellites count within *Sesamum* and *Ceratotheca* species chloroplast genomes


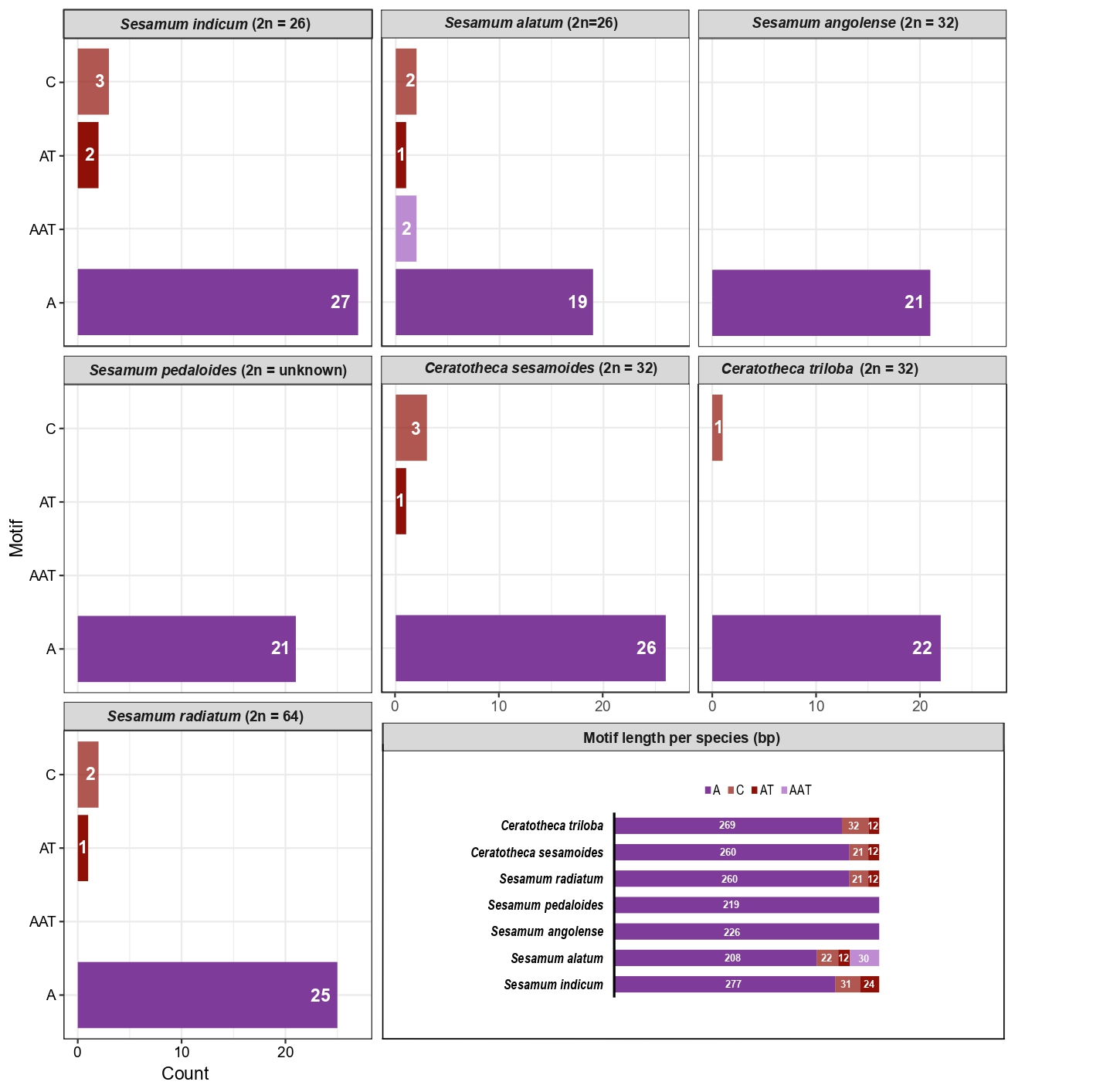


**Supplementary Figure S6.** Microsatellite motifs count within *Sesamum* and *Ceratotheca* species chloroplast genomes.
